# The role of healthcare professionals’ communication in trial participation decisions: a qualitative investigation of recruitment consultations and patient interviews across three RCTs

**DOI:** 10.1186/s13063-024-08656-y

**Published:** 2024-12-18

**Authors:** Nicola Farrar, Daisy Elliott, Marcus Jepson, Bridget Young, Jenny L. Donovan, Carmel Conefrey, Alba X. Realpe, Nicola Mills, Julia Wade, Eric Lim, Robert C. Stein, Fergus J. Caskey, Leila Rooshenas

**Affiliations:** 1 Population Health Sciences, University of Bristol, Canynge Hall, 39 Whatley Road, Bristol, BS8 2PS UK; 2 NIHR Bristol Biomedical Research Centre, University Hospitals Bristol and Weston NHS Foundation Trust and University of Bristol, Bristol, UK; 3 Department of Public Health, Policy and Systems, Institute of Population Health, University of Liverpool, Liverpool, L69 3GB UK; 4 The Royal Brompton and Harefield NHS Foundation Trust, Sydney Street, London, UK; 5 National Institute for Health Research University College London Hospitals Biomedical Research Centre, London, UK

**Keywords:** Qualitative, Recruitment, Randomised controlled trials

## Abstract

**Background:**

Although the challenges of recruiting to randomised controlled trials (RCTs) are well documented, few studies have focused on the impact that the communication between recruiters and patients has on patients’ participation decisions. Recruiters are thought to influence patient decision-making, but the mechanisms by which this occurs are unclear. The aim of this research was to investigate how patients interpret and use the information conveyed to them by healthcare professionals (HCPs) in trial participation decisions.

**Methods:**

Three pragmatic UK-based multicentre RCTs were purposively sampled to provide contrasting clinical specialities. Data collection was integrated into each RCT, including audio-recordings of patient recruitment consultations and interviews with patients. Where possible, consultation audio-recordings were linked to interviews to explore how information communicated by recruiters was interpreted and used by patients during their decision-making. Data were analysed thematically, using the constant comparison approach.

**Results:**

Twenty audio-recorded recruitment consultations were obtained across the 3 RCTs, combined with 42 interviews with patients who had consented to or declined RCT participation. Consultation and interview data were ‘linked’ for 17 individual patients. Throughout the patient’s clinical pathway, HCPs (both those involved in the RCT and not) influenced patients’ perceptions of treatment need and benefit by indicating that they preferred a particular treatment option for the patient as an individual. Whilst patients valued and were influenced by information conveyed by HCPs, they also drew on support from other sources and ultimately framed RCT participation decisions as their own. Patients’ willingness to be randomised hinged on perceptions of whether they stood to benefit from a particular treatment and the availability of those treatments outside of the trial.

**Conclusion:**

This study supports the need for training and support for healthcare professionals involved throughout the clinical pathway of patients eligible for RCTs, as all healthcare professionals who interact with patients have the potential to influence their perceptions of treatments being compared in the trial.

**Trial registration:**

OPTIMA ISRCTN42400492. Prospectively registered on 26 June 2012.

Prepare for Kidney Care ISRCTN17133653. Prospectively registered on 31 May 2017.

MARS 2 ISRCTN44351742. Retrospectively registered on 5 September 2018.

## Background

Recruitment to randomised controlled trials (RCTs) is often challenging, with many failing to recruit to time or to target [1]. Poor recruitment can lead to a trial closing early, not reaching its target sample size, or requiring extensions to funding [2, 3]. RCT discontinuation wastes resources [4] and can cause enrolled participants distress or frustration given the failure to achieve study objectives [5]. Research that focuses on understanding and improving recruitment to RCTs is a priority [4, 5], and several interventions intended to improve recruitment have been designed. A systematic review of interventions to improve RCT recruitment [6] found just three that could be graded as having high certainty of evidence of effectiveness, according to the GRADE (Grading of Recommendations, Assessment, Development and Evaluations) tool [7]. Only two of these were found to increase recruitment: (i) using open, rather than blinded allocation RCTs and (ii) telephone reminders for those who do not respond to postal invitation [6]. Given the small number of evidence-based interventions shown to improve recruitment [6], research to understand recruitment from the perspectives of key stakeholders (e.g. patients and recruiters) involved in recruitment processes is a priority [8–13].

Previous research has explored patients’ views and experiences of RCT recruitment processes and participation [12, 14–17]. A 2020 qualitative evidence synthesis (QES) highlighted the complexity of factors influencing a person’s decision to participate in an RCT, including trial-related and personal factors, as well as the importance of communication of trial information, and the influences of other people, including healthcare professionals (HCPs) [12]. Communication of information about an RCT can occur during a discussion with a clinician, research nurse, or other trial personnel or in the form of written/visual information (e.g. patient information sheet, videos, websites). Houghton et al. concluded with high confidence that HCPs can influence patient decision-making due to the trust placed in them [12]. Research into HCPs’ communication has included studies using audio and video recordings of consultations to understand how the RCT is explained to participants and the influence of communication about clinical management options on patient decision-making [18, 19], highlighting how doctors express uncertainty to patients [19], and the importance of the quality and the quantity of communication between HCPs and patients [18].

The National Institute for Health and Care Research (NIHR)-funded ProtecT RCT was the first to provide feedback to recruiters to improve their practices, based on a combination of audio-recordings of recruitment discussions and patient interviews to understand the study presentation. Feedback led to improvements in recruitment [20, 21]. A subsequent synthesis of findings from six RCTs using similar methods elucidated details of the obstacles and challenges to recruitment [22], leading to the development of a complex recruitment intervention to optimise recruitment: the ‘QuinteT Recruitment Intervention’ (QRI) [23, 24]. The QRI is designed to unearth and address recruitment issues in RCTs, a key component of which concerns how the trial is communicated to potential participants and interpreted by them.

Several qualitative interview studies have shown that patients often leave clinical encounters with an impression that their clinician favoured a particular treatment for them [12, 25–28]. In a study of interviews with recruiters and analysis of their audio-recorded discussions with patients in QRIs across six RCTs, Rooshenas et al. (2016) revealed that despite clinicians’ intentions to convey equipoise, it was often undermined or overridden in recruitment encounters with statements favouring particular treatment options [29]. These communication challenges were apparent across all six RCTs [29]. As it was often not possible to decipher how patients were interpreting the RCT information presented in the patient–recruiter interactions, Rooshenas et al. recommended that further research should seek to link audio-recorded consultations with patient interviews, building on previous research that had previously used this approach in the context of paediatric trials [30–33]. The study reported here was designed to build further evidence through analysis of data from a patient’s recruitment discussions in tandem with data from the same patient’s interview (conducted after participation decisions have been made), collected in QRIs across several RCTs. The aim of this research was to investigate how patients interpret and use the information communicated to them by healthcare professionals (HCPs) in trial participation decisions.

### Methods

This study used mixed qualitative methods to investigate what is communicated to patients during their recruitment consultations and how patients interpret and use this information when they consider RCT participation. Data collection involved: (i) audio-recording consultations where the RCT was anticipated to be discussed with patients (‘recruitment consultations’) and (ii) semi-structured qualitative interviews with patients following recruitment consultations [34]. Sampling for interviews and recruitment consultations took place from three UK-based, multicentre, phase III ‘host RCTs’, all of which were recruiting from secondary care, non-emergency settings and had integrated QRIs [23], which enabled data collection as part of the first author’s doctoral research. Approval for this work was granted from the London—Camberwell St. Giles Research Ethics Committee (reference: 13/LO/1481) (MARS 2), London—Surrey Research Ethics Committee (reference: 12/LO/0515) (OPTIMA), and South Central-Berkshire Research Ethics Committee (reference: 17/SC/0070) (Prepare for Kidney Care) and was secured at the ethical approval application stage for each RCT.

### Sampling of RCTs and patients

#### Sampling of RCTs

A portfolio of eight RCTs with integrated QRIs were open to recruitment in April 2018. The RCTs for this study were purposefully sampled based on an intention to include a range of disease areas and patient characteristics (e.g. age, sex) [35, 36]. The three sampled RCTs were all anticipated to have recruitment challenges from the outset. A summary of each RCT is provided below for context, with further details presented in Table 1.

**Table 1:** RCT characteristics and numbers of consultation audio-recordings and interviews

	RCT 1 (MARS 2)	RCT 2 (OPTIMA)	RCT 3 (Prepare for Kidney Care)	Total data collected across RCTs
Specialties involved	Oncology/respiratory/surgery	Oncology	Renal medicine	
Clinical context	Mesothelioma	Breast cancer	Kidney disease	
Interventions	1. Chemotherapy 2. Surgery and chemotherapy	1. Test directed chemotherapy 2. Chemotherapy	1. preparation for Responsive Management 2. Prepare for Renal Dialysis	
Number of (audio-recorded) recruitment consultations	13 (one patient had three recordings)	4 (one patient had two recordings)	3	20
Number of interviews (N with linked audio-recording)	21 (11)	16 (3)	5 (3)	42
Total number of linked audio-recordings and interviews	17			

The ‘MARS 2’ study, now complete, recruited patients with mesothelioma—a life-limiting cancer of the lung lining, predominantly caused by exposure to asbestos. Currently, the only treatment with evidence of improving survival is chemotherapy [37]. Surgery (extended pleurectomy decortication [EPD]) is not recommended outside a clinical trial [38]. Patients participating in MARS 2 were randomised between chemotherapy or chemotherapy plus surgery (EPD) [39].

The OPTIMA study is currently open and recruiting patients at high risk of recurrence from hormone-sensitive breast cancer who ordinarily receive chemotherapy in current practice. Patients are randomised between chemotherapy or ‘test-directed’ chemotherapy, where multi-parameter genomic testing is used to guide chemotherapy provision [40]. As such, some patients in the test-directed arm receive chemotherapy, and some do not.

Prepare for Kidney Care, also currently open, is recruiting patients with low kidney function who are either aged 80 years and above or 65 years and above with co-morbidities. Current practice varies, with some patients having dialysis and others ‘conservative care’ without dialysis. [41]. Patients are randomised between ‘preparation for Responsive Management’ (a study-specific form of conservative care) or ‘preparation for Renal Dialysis’ [42].

#### Sampling within RCTs

Sampling decisions were made in relation to data collection within each of the host RCTs. Although the original intention was only to collect data when both a consultation audio-recording and linked patient interview could be obtained, it became clear that interview data alone were still beneficial for addressing the study objectives due to the topic guide’s specific focus on how information from the recruitment consultation(s) was interpreted and used in decision-making about trial participation. As such, we decided to proceed with interviewing patients for whom there was no accompanying audio-recorded consultation. Given the anticipated difficulties collecting consultation recordings, such as logistical concerns and reluctance of clinicians to audio-record [43], no sampling criteria were placed on the collection of these data. A purposeful approach was taken with regard to patient interviews, with the intention of building a sample of male and female interviewees, and a mix of individuals who had consented to the RCT and individuals who had declined RCT participation.

### Data collection

#### Audio-recordings of recruitment consultations

Patients who were eligible to take part in each of the RCTs were invited to have their consultations audio-recorded by a member of the local clinical/trial team (e.g. research nurse, consultant) who was involved in their care. Information about the audio-recording process was provided via either the main RCT patient information sheet or an information sheet specific to the QRI. Written informed consent to audio-record was obtained from the local clinical/trial team.

#### Interviews with patients

Interviews were conducted by NF, a doctoral researcher with no previous association with the patients. All potential participants were informed about the interview process via an information sheet that explained both the audio-recording process and interview. Written informed consent for the interview was either obtained by the local site staff or lead author (NF) prior to the interview starting. A topic guide was used to explore patients’ recollections of their recruitment consultation, information conveyed to them about treatments and the RCT, and how they made their decision to accept or decline trial participation. The topic guide provided structure, whilst allowing new topics of discussion to be identified. The guide evolved and was revised throughout the course of data collection, to explore new lines of enquiry that developed from the concurrent analysis. Modifications to the wording and order of questions were also made, based on reflections from previous interviews and discussions with the wider team.

#### Data analysis

**Fig. 1:**
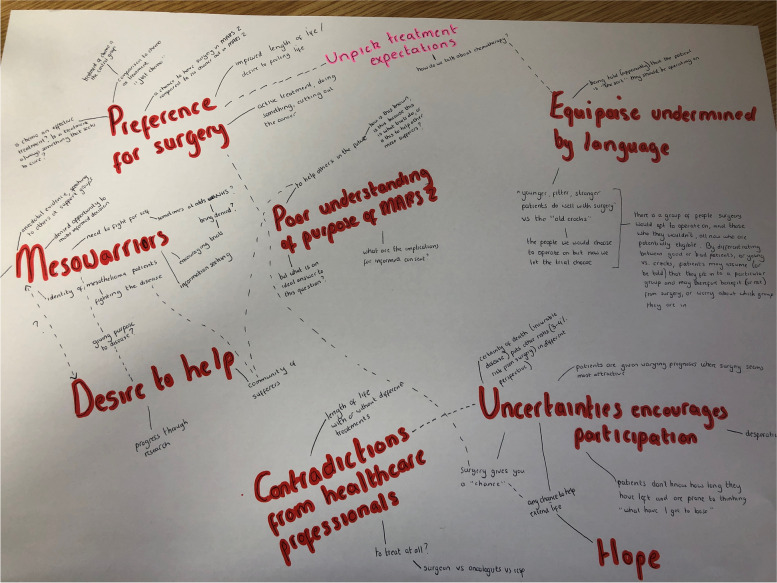
Theme map

All audio-recordings of recruitment consultations and interviews were transcribed by NF, a University of Bristol research administrator, or a paid transcription service. Upon receiving the transcripts, NF listened to the recording to check for errors/discrepancies. Transcripts were imported into NVivo V12 [44] where they were line-by-line-coded and analysed thematically [45], using the constant comparison method [46]. Initially, interview and consultation transcripts were analysed individually, and then, where possible, interviews and consultations with the same patient were linked for analysis, creating ‘cases’ [34]. This involved iterative comparisons between what was discussed during the consultation with how the patient interpreted topics identified through analysis of the consultation data (e.g. uncertainty, randomisation, and the treatments available) as evidenced during the consultation and the interview. During the initial stages of data collection, interview and consultation transcripts were shared and several were double-coded (LR, DE, and MJ) and discussed with members of the study team (LR, MJ, DE, and JD). Saturation was conceptualised according to the ‘inductive thematic approach’, focusing on the extent to which new codes or themes were identified in the data [47]. Descriptive accounts were drafted for each of the three RCTs individually and included both linked consultation and interview data, and ‘interview only’ data. Following analysis of data from each RCT independently, data and interpretations were compared across the three RCTs. This involved re-familiarisation with the entire dataset, exploring the main themes, sub-themes, and coded data in NVivo. From there, mind maps (Fig. 1 in the Appendix) were used to visualise key components of the analysis of each RCT and develop overarching themes that encapsulated relevant subtleties from each RCT.

## Results

This paper reports findings from an analysis of 20 audio-recorded recruitment consultations and 42 patient interviews. Seventeen audio-recordings were linked with a patient interview. The full overview of the data collected is reported in Table 1. Audio-recorded consultations took place in person between November 2018 and October 2020 and ranged from 9 to 90 mins. Interviews were collected between August 2018 and December 2020 via the telephone (*n* = 39) or face-to-face (*n* = 3), lasting between 16^1^ and 116 mins, with an average duration of 42 mins, and a median duration of 37 mins. Consultation audio-recordings were collected from eight sites. Interviews were undertaken with patients from 17 sites. Relatives and/or carers were often present and made contributions during consultations and interviews.

Findings are presented with illustrative quotations throughout. Quotations attributed to patients indicate the RCT they were approached about and whether they consented or declined participation. All participants (HCPs and patients) have been given pseudonyms to ensure anonymisation. Throughout data analysis, negative cases were explored and have been discussed, where relevant.

### Formation of early expectations of need and benefit

Patients from all three RCTs reported forming early expectations about treatments, based on discussions with HCPs that spanned the period from diagnosis to the first mention of the RCT. The length of time between these points in the pathway varied across individuals’ accounts and between the RCTs, but patients consistently discussed speaking to HCPs about their diagnosis, prognosis, and treatment options during this time. Patients considering participation in Prepare for Kidney Care described conversations with renal doctors and nurses about management approaches over the course of their declining kidney function. In their interviews, several patients reflected on their early discussions about treatment planning that focussed on one option, prior to learning about the trial:

 Interview with Mr Cole and partner (Prepare for Kidney Care study, decliner):

Mr Cole’s partner: “It was always put to him, he’d have the dialysis, and then we saw the nurse quite a few times didn’t we, and she showed us different things [methods of dialysing] and he decided which one he was going to have so it was set.”

Interviewer: “Was that a while ago, before they mentioned the study?”

Mr Cole: “Yeah yeah before they mentioned the study.”

OPTIMA patients often reported discussions about ‘standard care’ (chemotherapy) with surgeons and breast care nurses:

Interview with Ms Simpson (OPTIMA study, decliner):

Ms Simpson: “It was… the breast cancer nurse who told me [that the cancer was in the lymph nodes]. But from that, from that day I really thought that probably chemo would be the best option for me.”

Interviewer: “Was it the nurse that talked to you about chemo?”

Ms Simpson: “Yeah she told me what would, you know, what would happen. It would be sixteen weeks of chemo - no eighteen weeks is it? She explained what would happen.”

For MARS 2 patients, ‘standard care’ (chemotherapy) was reportedly established as the only evidence-based treatment available:

Interview with Mr Oliver (MARS 2 study, consenter):

Mr Oliver: “Two weeks later I saw the original doctor I’d seen who gave me the diagnosis. Basically it went something like - - "I’m sorry, you’ve got mesothelioma. Surgery isn’t an option for treatment. Radiotherapy doesn't work. The only treatment we can give you is chemotherapy which is palliative. There are no trials.".”

OPTIMA and MARS 2 included a treatment or approach that was not routinely used outside of the RCT. OPTIMA patients reflected in their interviews that chemotherapy was the established treatment for their disease type, and often, this understanding was attributed to information provided by HCPs prior to OPTIMA being introduced. This contributed to perceptions that RCT participation could lead to missing out on treatment if allocated to the ‘test-directed chemotherapy’ arm: an outcome that was not acceptable to some individuals who had come to understand that they needed, or would benefit from, chemotherapy:

Consultation between Ms Green (OPTIMA study, decliner), her partner and the Clinical Trials Officer:

Ms Green: “I mean everyone we have seen from the start to here have said, “Surgery. Chemo. Radiotherapy.” It’s bang, bang, bang.”

Interview with Ms Green (OPTIMA study, decliner):

Ms Green: “Basically all I heard in my head and then I switched off was, "You might not need chemotherapy…”. I didn’t really absorb much because, in my head, everyone had told me I’d needed chemotherapy, even the Macmillan nurses, because of the outcome of the lymph nodes. …Everyone had said that I would need it so I switched off at that point.”

Patients’ interview accounts revealed the importance of discussions with HCPs that took place before the RCT was described in detail, in building perceptions about whether a treatment was needed or potentially beneficial.

### Presentation and understanding of uncertainty

In all three RCTs, analysis of recruitment consultations showed some recruiters conveying equipoise to patients, primarily through articulating uncertainty about the best course of treatment and presenting a balanced description of the options available. In all RCTs, uncertainty was presented regarding the effectiveness of treatments, in terms of the primary outcomes for these trials:

Consultation with Mrs Connolly (MARS 2 study, consenter):

Respiratory01: “We don’t know whether it [surgery] works or not... We can’t promise you an operation, let alone promise that the mesothelioma will be cured by it, so you mustn’t go into this study thinking that either of those things are a distinct possibility.”

Consultation with Mrs Edwards (Prepare for Kidney Care study, decliner):

Research Nurse01: “And the problem we have is that, for patients who are over 65, if they have other health problems, or if they’re over 80, regardless of any other health problems, we don’t actually know which is best, as to whether you have a better quality of life or length of life if you choose to dialyse or if you choose not to dialyse and choose to manage your symptoms medically, if you like.”

Consultation with Ms Black (OPTIMA study, decliner):

OPTIMA_Oncolgoist02: “The idea of chemo is to try to kill or mop-up those stray cells…The problem is… we don’t know whether you’re someone that actually would benefit from chemo. So it could be that at the minute your cancer has gone. It’s never going to cause any problems or the hormone therapy will be enough. And we could potentially be giving you chemo, putting you through all that, for no good reason.”

Some recruiters, despite articulating uncertainty, also went on to override or undermine the position of equipoise, terms first introduced by Rooshenas et al. [29]. Overriding equipoise (as indicated in Ms Jones’ consultation below) refers to clinicians recommending a treatment option, often indicating that certain treatments would be more (or less) suitable for the patient as an individual, by virtue of their specific clinical characteristics or test results:

Consultation with Ms Jones (OPTIMA study, decliner):

OPTIMA_Oncolgoist01: “Now, usually we offer patients chemotherapy at this stage. We say to patients, “Your cancer was slightly big, your cancer was in the lymph nodes, your cancer was the type of cancer which tends to grow quickly.” For a cancer like your cancer, we don’t usually give patients a choice. We say to patients, “You should have chemotherapy.”…. I mean, I’ll feel very anxious if we don't give you chemotherapy.”

‘Undermining’ equipoise can be more subtle and is characterised by imbalanced presentation of treatment options, such as by using ‘loaded’ terminology to describe a study arm. In several extracts from MARS 2 consultations, the clinician explains that there is uncertainty over whether performing the operation is beneficial compared to not operating, but then reveals their impression that the patients are likely to benefit from surgery on the basis of being ‘young’ and ‘fit’. The surgeon also presents the outcome of surgery as looking ‘fantastic’ in the ‘young’ and ‘fit’ group:

Consultation with Mr Daniels (MARS 2 study, consenter):

MARS 2_Surgeon01: “When you compare the outcomes on those that we’ve operated on compared to those that we’ve not operated on, the results of surgery look fantastic. Now is that because the surgery works or is it because we’re good at choosing the winners?... I know what the results are in the younger fitter stronger patients that I tend to operate on. What I don’t know is whether it’s better than not having an operation..”

[Later]

“I might choose [to operate on] the young fit strong ones that I like the look of, like you, and I might, I might not choose others and therefore that again puts bias in so we need to make sure that we have an equal number of people like you in either side of the study.”

When interviewed, patients from all RCTs described reflecting on discussions to identify what they perceived as clinician recommendations. For example, following the OPTIMA consultation above, Ms Jones was clear that chemotherapy was ‘right’ for her, and Mr Daniels took reassurance from his discussion about his level of fitness as to the likelihood of better outcomes from the surgery:

Interview with Ms Jones (OPTIMA study, decliner):

Ms Jones: “He [Oncologist01] said it [chemotherapy] was the right thing for me.”

Interview with Mr Daniels (MARS 2 study, consenter):

Mr Daniels: “He [the surgeon] did give us a bit of encouragement, as to you know the level of fitness I still have, and had at the time of the appointment with him, that I was probably on the better side of you know some of the other patients you know had been diagnosed with the condition. I did think about it, and I weighed up you know the pros and cons and I was thinking to myself well how much could, do you generate without the surgery in six months and I thought well, one would probably outweigh the other, which I thought you know, was in the favour of you know, surgery.”

Patients’ perceptions of having received individual recommendations apparently played an important role in their decision-making about RCT participation. Many patients reported coming away from their consultation with the impression that a HCP had conveyed information about their individual suitability for a particular course of treatment or management, with suitability often associated with a potential need for, or benefit from, that treatment:

Interview with Mr Sutherland and partner (MARS 2 study, consenter):

Mr Sutherland’s partner: “He [surgeon] just said that you were ideal for it [surgery].”

Interview with Ms Campbell (OPTIMA study, decliner):

Ms Campbell: “So, I was really glad when he [oncologist] was open and honest and said ,‘look you know, it’s not worth you going through that [chemotherapy] for this 2% [anticipated benefit of chemotherapy].’ So, he even swerved me from it [chemotherapy], which I found really fair.”

### Participation as a considered and individual decision

Whilst some patients framed their perceptions of what treatments they needed or would benefit from around HCPs’ recommendations, others described being more reliant on their own assimilation of information. They framed their RCT participation decisions as inherently individual decisions they had made themselves:

Interview with Ms Miller (OPTIMA study, decliner):

Ms Miller: “I can understand why they’re going for it [OPTIMA], and it probably does sound a bit selfish, but in these situations, you think about your own life and you think, “I don’t want to go through something if it isn’t going to make any difference”.”

Interview with Mr Owen (MARS 2 study, consenter):

“I need to make a decision that’s better for me, and weigh up the pros and cons.”

Patients often described a process of ‘weighing up’ information about the potential risks and benefits to their own health and altruistic motives of improving the health of others. For OPTIMA and Prepare for Kidney Care patients, risks of RCT participation were often framed in terms of not receiving treatment they perceived to be necessary or beneficial and which would have been open to them outside of the RCT:

Interview with Mr Cole (Prepare for Kidney Care study, decliner):

Mr Cole: “Don’t get me wrong I don’t want dialysis, but, you know if I need something then I think I’d rather have the dialysis than, than not have it if you know what I mean.”

Interview with Ms Grant (OPTIMA study, decliner):

Ms Grant: “So then it was as if the OPTIMA could be something that would deny me the treatment [chemotherapy] I thought I needed to have.”

In contrast, most patients approached about MARS 2 offered a different view on risk, with several perceiving that they had ‘nothing to lose’ by entering the study given their life-limiting illness and the perceived lack of availability of surgery outside the RCT:

Interview with Mr Oliver (MARS 2 study, consenter):

Mr Oliver: “I know it’s not a cure but I’m looking at, hopefully, as I said before, clawing back [through surgery] some of those years I’m going to lose. Before that I didn’t have any hope.”

As well as the risks, patients often reflected on the potential benefits of RCT participation, including benefits for their own health:

Interview with Mr Stanley (MARS 2 study, consenter):

Mr Stanley: “I’ve read everything and everything they’ve told me about how long I might live with just chemo, how I might feel… I just feel for me, I feel, I want to give myself every chance [with surgery].”

Interview with Mrs Harrison (OPTIMA study, consenter):

Mrs Harrison: “Right at the end when they said about this [OPTIMA], but they were finding people having chemotherapy that didn’t actually need it and this trial - and they explained it all to me and I thought, “well I’m going to go for that”, I’ve got nothing to lose. This is such an amazing thing to be offered.”

Across all the RCTs, patients indicated how they had considered that RCT participation contributed towards advancing knowledge that could help future patients with their condition:

Interview with Mr Eastlake (Prepare for Kidney Care study, consenter)

Mr Eastlake: “I mean, at the end of the day as well, if it [RCT participation] helps generally, on a decision that doctors have to make…in the future for other people, I’m quite happy”

Regardless of whether patients accepted or declined the RCT, helping others in a similar situation was viewed positively and several who declined the RCTs still reportedly wanting to contribute to research. Several who declined the RCTs indicated that they chose to protect their own interests first—the perceived risk of not being allocated to a treatment they understood to be beneficial, or being exposed to a treatment they considered could be harmful, overrode their altruistic motivations.

### The supportive role of family and friends

Friends and family were reported to play a role in the decision-making process, in particular when they had an experience of or connection to the condition or healthcare more widely. Often, they had a more confirmatory or supportive role in decision-making, with patients reiterating that their decision was their own:

Interview with Mr Harper and partner (MARS 2 study, decliner):

Mr Harper’s partner: “Our son, his wife works at [hospital], so we’ve had quite a lot of insights, including from [daughter in law]. Yeah, we’ve had completely open discussions with them, yes discussions, they’ve not told us what to do, just talked about what they’ve found and so on. The decision’s been ours entirely. She was very against the surgery, because having worked in orthopaedics and now working as a district nurse, she’s well aware of the after effects.”

Interview with Ms Black (OPTIMA study, decliner):

Ms Black: “I have a very good friend who’s a professor of cancer. She's a nurse professor. And she's at terminal stage, unfortunately she has breast cancer and it came back after ten years… but I chatted to her quite a bit, just to understand it because she spent her whole life in research, with cancer.

[Later]

“To be honest my husband’s an accountant he’s not a scientist so he was very much, you know, it’s up to me.”

Patients often reported feeling overwhelmed by information, with several struggling to take in their diagnosis and prognosis. Several patients reported missing important information during their interactions with HCPs:

Interview with Mr Goodwin (MARS 2 study, consenter)

Mr Goodwin: “I don’t know if you’ve ever found yourself in a situation like this but you go along and people are talking to you and it’s like firing bullets at a wall, most of them bounce off, so it’s handy to have someone with you, who can also listen to and remind you of things that you certainly do miss in general conversation.”

Friends and family therefore often played a supportive role, reminding patients of information they may have missed and discussing again what they were told during the consultation. Several MARS 2 and OPTIMA patients discussed using the internet to gain additional information about their condition and potential treatment options, generally using it as an adjunct to the information provided to them by their HCPs:

Interview with Ms Harrison (OPTIMA study, consenter)

Ms Harrison: “They [HCPs] made sure that I had time to ask questions if I had any. Of course I'm an inveterate looker-up-on-the-internet so will look into every why, wherefore and how. Just to get all the relevant information. And, you know, I go to reputable sites not scare mongering…Just to get any information if I - there weren't really very many questions that I had because it was explained so well to me in the visits I had.”

### Randomisation as a barrier and enabler to accessing treatments

Whether patients perceived that they would benefit from, or needed, a particular treatment shaped whether they accepted randomisation. When patients believed a specific treatment was needed or beneficial, they rejected the RCT on the basis that randomisation could deny them the option to access that treatment—especially if that treatment could be accessed outside of the RCT. Conversely, in MARS 2, several patients accepted trial participation (sometimes reluctantly) as a way of potentially accessing surgery that would not be available to them outside of the trial:

Interview with Mr Heath (MARS 2, consenter):

Mr Heath: “I said [to surgeon] is there any way you can operate on me without being involved in this MARS 2 thing <laughs> he said not a chance <laughs>.”

Where trial participation was deemed to potentially deny access to the preferred treatment, patients indicated that the idea of randomisation caused distress, frustration, and made little sense. When explored in interviews, this emotional response to randomisation stemmed from an understanding that it risked them receiving what they understood to be an inferior and/or sometimes unnecessary treatment:

Interview with Mr Cole (Prepare for Kidney Care study, decliner):

Interviewer: “How did you feel about the idea of that [randomisation]?”

Mr Cole : “Not very keen…not very keen for the simple reason that…dialysis seems to be the way to go, everything has been geared up for that and [I wasn’t] too happy about taking the chance on not having dialysis.”

Interview with Ms Anderson (OPTIMA study, decliner):

Ms Anderson: “Because they said “it's a trial you would go into”, and I thought: “okay what does that mean?” And it's basically, there's lots of people in it, and you're picked at random by what treatment you have… So I thought: “wow, that doesn't sound great to be honest. So what do you mean by that?” So she said, “well, they will decide what treatment you have and some people don't get any treatment, but some people do”. And I said: “oh my God no.” Like, you know, I've got three children, I'm not gambling my life. So, no definitely not.”

Some MARS 2 and OPTIMA patients expressed a desire for a method other than randomisation to be used for treatment allocation but nonetheless acknowledged that this was the route they had to go down to get a chance of receiving their preferred treatment:

Interview with Mr Oliver (MARS 2 study, consenter):

Mr Oliver: “Well, I'd much rather know that I'm going to get the surgery but I've got a 50% chance of getting the surgery as opposed to having no chance at all if I don't go on the trial.”

Interview with Ms Clark (OPTIMA study, consenter):

“I wish it hadn’t been totally random…but at the end of the day that was how it worked, so I just had to accept that.”

## Discussion

This study reports findings that unpack how patients understand and apply information provided to them in RCT participation decision-making, across three RCTs in different clinical areas. The information conveyed by HCPs played a central role in decision-making about trial participation, primarily through shaping patients’ perceptions of what treatments they needed or stood to benefit from. These perceptions of need and benefit were often formed early on through clinical discussions that preceded the presentation of the RCT. Although many recruiters tried to articulate equipoise by stating the uncertainty as to the best treatment for the patient, several subsequently overrode this through explicit recommendations or undermined equipoise through imbalanced presentations. Whilst patient decision-making about RCT participation was often rooted in perceptions of need and benefit that were informed by HCPs, many patients expressed ownership of their participation decisions. As part of the decision-making process, patients’ families and friends were often involved, especially if they had a medical connection, often in a supportive or confirmatory role. Whether patients accepted randomisation or not was linked to perceptions of their needs and capacity to benefit, in the context of whether treatments were available outside of the RCT.

Audio-recorded consultations and patient interviews analysed in this study indicated that information provision from HCPs shaped patient understanding of what treatments they would–and should-have. This accords with prior evidence which highlighted how HCPs are in a position of influence when patients make decisions about RCT participation [12] and how patients often leave clinical encounters with a belief that their clinician had favoured a particular treatment for them [12, 25–28]. This study adds insights into the role of HCPs outside the recruiting team in contributing towards perceptions that a particular treatment is needed or beneficial for the patient, often based on the patients’ clinical characteristics. These perceptions, established prior to the introduction of the RCT, can then be challenging for recruiters to overcome. These data also confirm findings from QRIs about how important it is that patients receive clear and balanced information about the RCT, its trial arms, and the purpose of randomisation from HCPs if they are to make fully informed decisions about trial participation [22, 29, 48–50].

Recruiters face many intellectual and emotional challenges when communicating equipoise [51], and previous work by Rooshenas et al. highlighted how challenging it can be for clinicians to communicate equipoise to patients [29]. The latter introduced the idea that equipoise is often overridden or undermined through treatment recommendations or imbalanced presentation of treatments. The present study extends this by showing how this reduced the opportunities for patients to fully understand that the uncertainty around treatment benefits extended to them. Based on our analysis of consultation recordings here, recruiters at times overrode or more often undermined equipoise in discussions with patients, which was often confirmed in patient interviews. In explaining the uncertainty underpinning the RCT, recruiters sometimes countered these explanations with suggestions that individual patients could derive benefit from particular treatments. The study has also shown how even subtle undermining of equipoise can lead to patients perceiving treatment recommendations, and this can happen with staff at any point in the patient’s pathway. The notion that both recruiting [16, 25, 52–54] and non-recruiting [55–57] members of staff can have preferences for patients to receive particular procedures or treatments is well-established, but our study has shown the direct implications for RCT participation decisions, in that patients retained and drew heavily upon their interpretations of HCPs’ recommendations. Ultimately, although recruiters may believe in the concept of equipoise, they may find it difficult to maintain a position of equipoise when faced with an individual patient about whose treatment they have strong beliefs [51].

Patients in this study considered participation a personal decision, in which they considered the trade-offs involved in participating in the RCT, weighing up perceived risks and benefits. Risks of RCT participation were generally considered in the context of access to perceived necessary/beneficial treatments. Patients also considered how the RCT could help themselves, as well as others, a phenomenon reported as ‘conditional altruism’ [58]. The idea that patients ‘weigh-up’ their decisions to participate in RCTs is widely discussed and is based on research in several different populations and clinical areas [12, 16, 17, 25, 58–67]. The findings reported here highlight how recruiters play a role in shaping interpretations of treatment benefit and need, which in turn can contribute towards perceptions of risks and benefits with study participation.

Whilst patients relied heavily on the verbal information communicated to them by HCPs, they often sought further support elsewhere, as found previously by Houghton et al. who found that patients regularly discuss study participation with a range of people, as well as consulting internet/media sources [12]. In this study, friends and family, particularly those with a healthcare background, were often called upon to affirm decisions about treatment and RCT participation, whilst internet sources were used for further information, with participants often recognising the risk of sourcing information from unreliable websites. Further research could helpfully explore whether the nature of the condition, treatments available, and timings of appointments impact the extent of any online research undertaken prior to discussions with recruiters.

Concerns about limits on access to treatments perceived necessary or beneficial often manifested in a rejection of randomisation. Where patients agreed to take part in the RCT, some did so as it provided them the possibility of accessing the treatment they felt would be beneficial, although several were aware there were no guarantees of receiving their preferred treatment arm. Interviews with patients approached to take part in the ProtecT study found that agreement to be randomised was closely related to patient equipoise and understanding of the rationale for randomisation [50, 68]. Patients’ views on randomisation were rarely expressed during consultations, which highlights an opportunity for recruiters to explore patients’ views as part of the recruitment process.

This study has highlighted the influence that the patient’s pathway can have on their eventual recruitment, as perceptions of need and benefit can be established early on. It is important to understand the importance of clinical encounters outside those traditionally considered to be part of ‘recruitment appointments’ [23, 69] on patient decision-making. In the UK, there is currently a government-led drive to integrate research into the heart of patient care within the NHS [70]. There is also clear support to do this from the research community, who identified the question “How can randomised trials become part of routine care and best utilise current clinical care pathways?” as the leading priority during the 2018 James Lind Alliance Prioritising Recruitment In Randomised Trials study [5]. Future interventions to optimise RCT recruitment should consider the role of personnel throughout the patient’s care pathway, including individuals who may not identify as ‘recruiters’. Future studies embedded in RCTs should ensure that they explore and understand these individuals’ views and beliefs about the trial when considering the development of training and recruitment support materials. This research also highlights the potential value in exploring patients’ perceptions of treatments early on in the recruitment discussion in the interests of facilitating informed decision-making [50].

Improvements in communication about an RCT may impact on levels of patient understanding [50], but may not necessarily translate into an increase in numbers recruited, despite improving understanding for informed consent. Work around measuring informed consent and RCT deliberation is underway [71, 72].

### Strengths and limitations

Strengths of the study lie in the linkage of patient interviews and recruitment interactions across a range of RCT contexts. This added value to the research as it enabled us to explore how patients interpret the information they were provided. As an example, with interviews alone, we may have assumed that HCPs often simply provided treatment recommendations to patients. However, with the recruitment interaction data, we were able to highlight the complex nature of conveying equipoise, with HCPs often attempting to express equipoise, sometimes in spite of personal beliefs. The triangulation of findings through constant comparison, across three very different RCTs, allowed this research to generate findings that will likely be broadly applicable to a wide range of RCT contexts. The RCTs from which data were collected and analysed were all phase III, pragmatic RCTs in non-emergency secondary care settings, with adult patients who were able to consent for themselves. The experiences of the patients in this sample may not be transferable to different types of research, earlier phase trials, research conducted in emergency settings, or where the patient may not have the capacity to consent for themselves. All three of the RCTs included in this study could be considered to be in ‘high-stakes’ settings due to the very different lifestyle implications of the treatments being compared, and/or implications for survival, and given that all three of the RCTs concerned potentially life-limiting diseases. The significance of healthcare professionals’ communication of equipoise, and how this is interpreted, was important in patients’ decision-making in these contexts, but it is plausible that these communication issues play a less salient role in decision-making where the stakes are less high. Repeating this work in a wider array of RCTs would be valuable for examining the weighting of clinician communication on patients’ decision-making about trial participation and treatments. Demographic details were not collected on the participants, making it difficult to comment on the transferability of the findings based on socio-demographic characteristics. RCTs themselves are also rarely representative of the target population [73]. There is a need to address our research question with a specific focus on patient groups that historically have been more or less likely to take part in RCTs. For example, there is a known need for more research to examine the factors that affect the recruitment of black, Asian, and minority ethnic groups to RCTs [12].

## Conclusion

The findings of the research published here indicate that in order to successfully integrate research into routine care, staff with patient-facing roles may have to reconsider how they communicate key topics to patients, so as to avoid constructing perceptions of need or benefit in the context of equipoise and ongoing RCTs. This study indicates the need to extend the reach and focus of recruitment interventions (such as the QRI, from which the data in this study were drawn) to be inclusive of individuals responsible for presenting the trial to potential participants, as well as those throughout the clinical pathway who may have a more peripheral association with the RCT.

## Data Availability

The supporting data underpinning this research are ‘controlled data’. Details of how to request access are available via the University of Bristol: https://www.bristol.ac.uk/staff/researchers/data/accessing-research-data/.

## Footnotes

1 One interview (duration 7 min) was terminated early due to participant distress.
